# Endosomal trafficking protein TBC-2 is required for the longevity of long-lived mitochondrial mutants

**DOI:** 10.3389/fragi.2023.1145198

**Published:** 2023-05-16

**Authors:** Annika Traa, Hazel Shields, Abdelrahman AlOkda, Zenith D. Rudich, Bokang Ko, Jeremy M. Van Raamsdonk

**Affiliations:** ^1^ Department of Neurology and Neurosurgery, McGill University, Montreal, QC, Canada; ^2^ Metabolic Disorders and Complications Program, Research Institute of the McGill University Health Centre, Montreal, QC, Canada; ^3^ Brain Repair and Integrative Neuroscience Program, Research Institute of the McGill University Health Centre, Montreal, QC, Canada; ^4^ Division of Experimental Medicine, Department of Medicine, McGill University, Montreal, QC, Canada

**Keywords:** aging, lifespan, stress resistance, endosomal trafficking, *C. elegans*, genetics, TBC-2, DAF-16 transcription factor

## Abstract

Mutations that result in a mild impairment of mitochondrial function can extend longevity. Previous studies have shown that the increase in lifespan is dependent on stress responsive transcription factors, including DAF-16/FOXO, which exhibits increased nuclear localization in long-lived mitochondrial mutants. We recently found that the localization of DAF-16 within the cell is dependent on the endosomal trafficking protein TBC-2. Based on the important role of DAF-16 in both longevity and resistance to stress, we examined the effect of disrupting *tbc-2* on lifespan and stress resistance in the long-lived mitochondrial mutants *nuo-6* and *isp-1* in *Caenorhabditis elegans*. Loss of *tbc-2* markedly reduced the long lifespans of both mitochondrial mutants. Disruption of *tbc-2* also decreased resistance to chronic oxidative stress in *nuo-6* and *isp-1* mutants but had little or no detrimental effect on resistance to other stressors. In contrast, *tbc-2* inhibition had no effect on oxidative stress resistance or lifespan in *isp-1* worms when DAF-16 is absent, suggesting that the effect of *tbc-2* on mitochondrial mutant lifespan may be mediated by mislocalization of DAF-16. However, this result is complicated by the fact that deletion of *daf-16* markedly decreases both phenotypes in *isp-1* worms, which could result in a floor effect. In exploring the contribution of DAF-16 further, we found that disruption of *tbc-2* did not affect the nuclear localization of DAF-16 in *isp-1* worms or prevent the upregulation of DAF-16 target genes in the long-lived mitochondrial mutants. This suggests the possibility that the effect of *tbc-2* on lifespan and stress resistance in the long-lived mitochondrial mutants is at least partially independent of its effects on DAF-16 localization. Overall, this work demonstrates the importance of endosomal trafficking for the extended longevity and enhanced stress resistance resulting from mild impairment of mitochondrial function.

## Introduction

Mitochondria are membrane-bound organelles that account for most of the energy production in the cell and have important roles in intracellular signaling and metabolism. Despite the importance of mitochondria to cellular function, mild impairment of mitochondrial function can extend lifespan and increase resistance to stress (Wong et al., 1995; Lakowski and Hekimi, 1996; Feng et al., 2001; Yang and Hekimi, 2010; Schaar et al., 2015; Dues et al., 2017; Wu et al., 2018). The ability of mutations affecting the mitochondria to extend lifespan has been demonstrated in multiple species including worms (Wong et al., 1995; Lakowski and Hekimi, 1996; Feng et al., 2001; Yang and Hekimi, 2010), flies (Copeland et al., 2009) and mice (Dell’agnello et al., 2007; Liu et al., 2005), thereby indicating conservation across species.

Among the most well studied mitochondrial mutants in *Caenorhabditis elegans* are *nuo-6* and *isp-1.* The *nuo-6* gene encodes a subunit of Complex I of the mitochondrial electron transport chain (Yang and Hekimi, 2010), while the *isp-1* gene encodes a subunit of the Rieske iron sulfur protein in Complex III of the electron transport chain (Feng et al., 2001). Although a number of studies have identified factors that are important for the long lifespan of these mitochondrial mutants (Lee et al., 2010; Walter et al., 2011; Baruah et al., 2014; Hwang et al., 2014; Yee et al., 2014; Munkacsy et al., 2016; Senchuk et al., 2018; Wu et al., 2018; Campos et al., 2021; Jung et al., 2021; Harris-Gauthier et al., 2022), the mechanism by which mild mitochondrial impairment extends longevity remains incompletely understood.

Among the factors that are required for the longevity of the long-lived mitochondrial mutants is the FOXO transcription factor DAF-16 (Senchuk et al., 2018). *nuo-6* and *isp-1* mutants exhibit increased expression of DAF-16 target genes, which is dependent on DAF-16 and the DAF-16 deubiquitylase MATH-33. DAF-16 is part of the insulin/IGF-1 signaling pathway. Under conditions of decreased insulin/IGF-1 signaling or increased stress, DAF-16 translocates from the cytoplasm to the nucleus to modulate gene expression. Disrupting DAF-16 reverts *nuo-6* and *isp-1* lifespan to wild-type (Senchuk et al., 2018).

We recently showed that cytoplasmic DAF-16 can be localized to endosomes (Meras et al., 2022). Disruption of the insulin/IGF-1 signaling pathway decreases endosomal localization of DAF-16 while increasing its localization to the nucleus. Endosomal localization of DAF-16 is also affected by disruption of endosomal trafficking proteins. Specifically, loss of the Rab GTPase activating protein (GAP) TBC-2 increases the localization of DAF-16 to endosomes, while inhibition of the TBC-2 targets RAB-5 and RAB-7 has the opposite effect (Chotard et al., 2010; Law and Rocheleau, 2017; Meras et al., 2022). The increased endosomal localization of DAF-16 in *tbc-2* mutants results in a decreased ability of DAF-16 to translocate to the nucleus in response to specific stresses (Traa et al., 2022). In addition, disruption of *tbc-2* reduces the upregulation of DAF-16 target genes in long-lived *daf-2* mutants and decreases their lifespan (Meras et al., 2022).

Under normal conditions, internalized vesicles containing cargo from the plasma membrane, such as ligand bound receptors, fuse together to form early endosomes, which contain RAB-5. From the early endosome, the internalized cargo can be sorted to the recycling endosome, the Trans-Golgi network or to late endosomes, which contain RAB-7. Cargo transported to late endosomes can then be degraded by the lysosome. When *tbc-2* is disrupted, there is an accumulation of enlarged late endosomes resulting from increased activation of RAB-5 (Chotard et al., 2010).

In this work, we examined the role of TBC-2 in the extended longevity and enhanced stress resistance of long-lived *C. elegans* mitochondrial mutants. We found that disruption of *tbc-2* decreases lifespan and resistance to specific exogenous stressors in *nuo-6* and *isp-1* worms. Surprisingly, the *tbc-2* deletion did not affect the increased nuclear localization of DAF-16 in *isp-1* mutants and failed to prevent upregulation of DAF-16 target genes in the long-lived mitochondrial mutants. Overall, this work demonstrates the importance of TBC-2 in stress resistance and longevity and suggests that *tbc-2* disruption may be affecting these phenotypes in long-lived mitochondrial mutants at least partially through DAF-16-independent pathways.

## Materials and methods

### Strains

N2 (WT)

QR15 *tbc-2(tm2241) II*

JVR171 *isp-1(qm150) IV*

MQ1333 *nuo-6(qm200) I*

JVR555 *isp-1(qm150) IV;tbc-2(tm2241)*

JVR556 *nuo-6(qm200) I;tbc-2(tm2241)*

JVR502 *daf-16(mu86) I;isp-1(qm150) IV*

JVR596 *daf-16(mu86) I;tbc-2(tm2241) II*

JVR619 *daf-16(mu86) I;tbc-2(tm2241) II;isp-1(qm150) IV*

JVR333 *daf-16(mu86) I;zIs356 [daf-16p::daf-16a/b::GFP + rol-6(su1006)] IV*

JVR640 *daf-16(mu86) I;isp-1(qm150) IV;zIs356 [daf-16p::daf-16a/b::GFP + rol-6(su1006)] IV*

JVR641 *daf-16(mu86) I;isp-1(qm150 IV; tbc-2(tm2241) II; zIs356 [daf-16p::daf-16a/b::GFP + rol-6(su1006)] IV*

Worms were maintained at 20°C on NGM plates seeded with OP50 bacteria.

### Heat stress assay

Resistance to heat stress was measured by transferring 25 pre-fertile young adult worms to freshly seeded NGM plates, incubating at 37°C and monitoring survival every 2 h for a total of 10 h.

### Chronic oxidative stress assay

Resistance to chronic oxidative stress was measured by transferring 30 pre-fertile young adult worms to 60 mm NGM plates containing 4 mM paraquat (methyl viologen, Sigma Catalog No. 856177) and 100 μM FUdR and seeded with concentrated OP50 bacteria (see (Senchuk et al., 2017) for detailed protocol). Worms were kept at 20°C and survival was monitored daily until all worms died.

### Acute oxidative stress assay

Resistance to acute oxidative stress was measured by transferring 30 pre-fertile young adult worms to 300 μM juglone plates seeded with OP50 bacteria and monitoring survival at 20°C every 2 h for a total of 8 h. Plates were made fresh on the day of the assay as juglone toxicity diminishes rapidly with time (see http://wbg.wormbook.org/2016/07/14/measuring-sensitivity-to-oxidative-stress-the-superoxide-generator-juglone-rapidly-loses-potency-with-time/).

### Osmotic stress assay

To measure resistance to osmotic stress, 25 pre-fertile young adult worms were transferred to 450 or 550 mM NaCl plates seeded with OP50 bacteria and survival was scored after 24 h at 20°C.

### Anoxia assay

To measure resistance to anoxic stress, 45 pre-fertile young adult worms were transferred to NGM plates freshly seeded with OP50 bacteria and sealed in low-oxygen Becton-Dickinson Bio-Bag Type A Environmental Chambers. After 48 h at 20°C, worms were allowed to recover outside of the chamber for 24 h before survival was measured.

### Bacterial pathogen stress assay

Resistance to bacterial pathogen stress was performed as previously described (Campos et al., 2021). 45 L4 worms were transferred to 100 mg/L FUdR plates seeded with OP50 bacteria and were grown at 20°C until day 3 of adulthood. Day 3 adult worms were then transferred from these plates to 20 mg/L FudR plates seeded with *Pseudomonas aeruginosa* (PA14). Worms were kept at 20°C and survival was monitored daily until all worms died.

### Lifespan assay

To measure lifespan, 50 pre-fertile young adult worms were transferred to 25 µM FUdR plates seeded with OP50 bacteria and kept at 20°C. Low concentrations of FUdR limit internal hatching and inhibit development of progeny without affecting longevity in wild-type worms (Van Raamsdonk and Hekimi, 2011). Survival was monitored daily until all worms died.

### Quantification of mRNA levels by quantitative RT-PCR

Quantitative RT-PCR was performed by collecting worms in M9 buffer. RNA was extracted using Trizol as we have described previously (Machiela et al., 2016) and converted to cDNA using a high-capacity cDNA Reverse Transcription kit (Applied Biosystems 4368814). Quantitative PCR was performed using a PowerUp SYBR Green Master Mix (Applied Biosystems A25742) in a MicroAmp Optical 96-well reaction plate (Applied Biosystems N8010560) and a Viia 7 applied biosystems qPCR machine. mRNA levels were calculated as the copy number of the gene of interest relative to the copy number of the endogenous control, *act-3*, and expressed as a percentage of WT. Primer sequences for each target gene are as follows:

*sod-3* (AAA​GGA​GCT​GAT​GGA​CAC​TAT​TAA​GC, AAG​TTA​TCC​AGG​GAA​CCG​AAG​TC), *dod-3* (AAG​TGC​TCC​GAT​TGT​TAC​GC, ACA​TGA​ACA​CCG​GCT​CAT​TC), *mtl-1* (ATG​GCT​TGC​AAG​TGT​GAC​TG, GCT​TCT​GCT​CTG​CAC​AAT​GA), *sodh-1* (GAA​GGA​GCT​GGA​AGT​GTT​GTT​C, CTC​CAC​GTA​TAG​TGA​GGT​ACT​CCT​G), *ftn-1* (GAG​TGG​GGA​ACT​GTC​CTT​GA, CGA​ATG​TAC​CTG​CTC​TTC​CA), *icl-1* (TGT​GAA​GCC​GAG​GAC​TAC​CT, TCT​CCG​ATC​CAA​GCT​GAT​CT), *act-3* (TGC​GAC​ATT​GAT​ATC​CGT​AAG​G, GGT​GGT​TCC​TCC​GGA​AAG​AA).

### Nuclear localization of DAF-16

DAF-16 localization to the nucleus was imaged in young adult hermaphrodite worms at the level of the whole body using the DAF-16::GFP reporter (zIs356[*daf-16p::daf-16::GFP*]) in *isp-1;daf-16* worms and *isp-1;daf-16;tbc-2* worms. We used worms with mutations in the endogenous *daf-16* gene to ensure that the DAF-16 being localized to the nucleus has the GFP reporter. Worms were mounted onto 2% agarose pads and immobilized with 5–10 μL of 10 mM levamisole. Images were obtained used a ZEISS LSM780 confocal microscope using a ×40 objective lens. Images were then analyzed on Fiji (ImageJ) where the number of GFP positive foci was determined using the particle analysis tool. Approximately 20 worms per genotype were imaged over 3 replicates.

### Statistical analysis

At least three biological replicates were completed in each experiment. For lifespan and stress assays, the experimenter was blinded to the genotype to ensure unbiased results. Worms were excluded from lifespan and stress assays if they crawled off the agar and died on the side of the plate, had internal hatching of progeny or expulsion of internal organs. Statistical significance of differences between genotypes was determined using a one-way ANOVA with Dunnet’s multiple comparison test, two-way ANOVA with Šidák’s multiple comparison test, a repeated measures ANOVA, or a log-rank test using Graphpad Prism. Complete statistical analysis can be found in Supplementary Table S1.

## Results

### Disruption of TBC-2 decreases the lifespan of long-lived mitochondrial mutants

**FIGURE 1:**
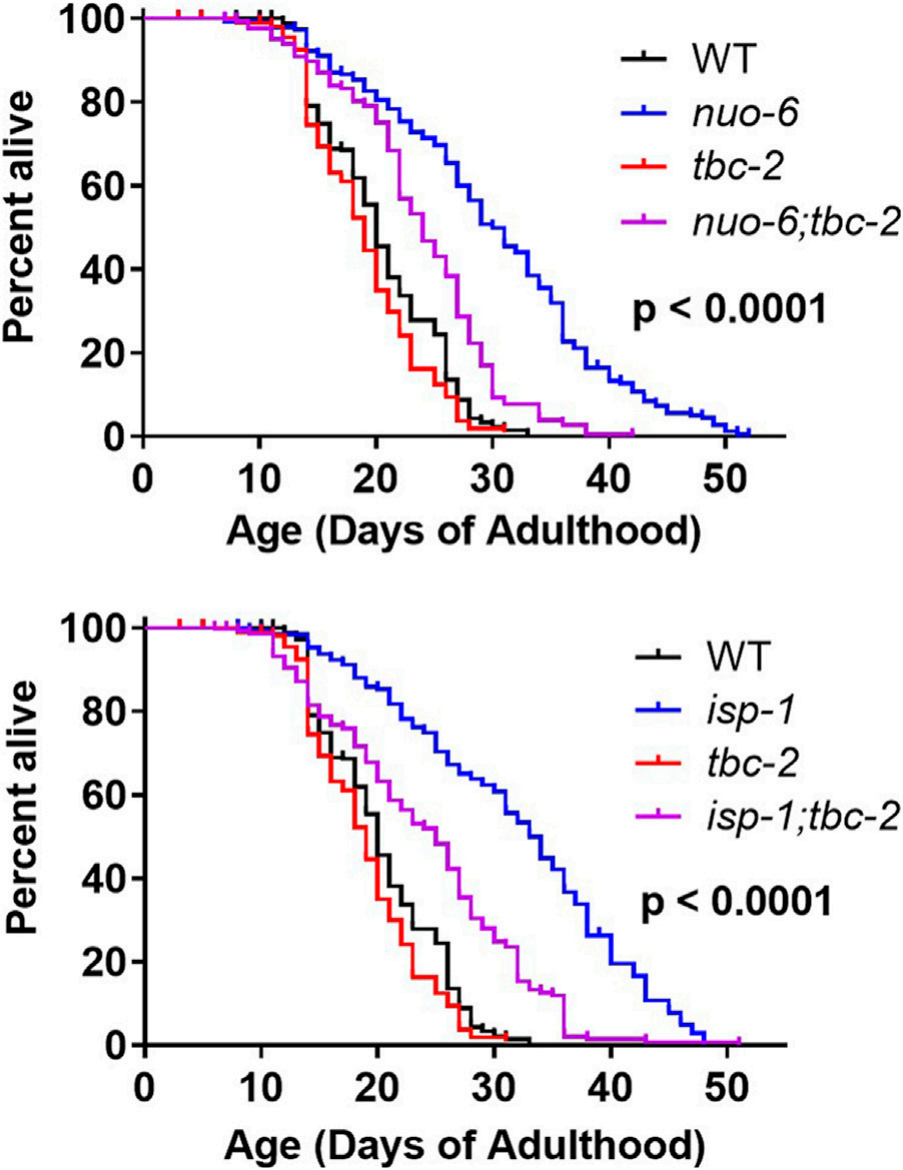
TBC-2 is required for the longevity of long-lived mitochondrial mutants. The long-lived mitochondrial mutants *nuo-6* and *isp-1* were crossed to *tbc-2* deletion mutants. Disruption of *tbc-2* markedly reduced the lifespan of both mutants, while only having a minor impact on wild-type lifespan. Significance indicates differences between blue and purple lifespan curves and was determined using the log-rank test. The Bonferroni-corrected threshold for significance is *p* = 0.00833. A minimum of three biological replicates per strain were performed. Raw lifespan data and full statistical comparisons can be found in Supplementary Table S1.

We previously showed that both *daf-2* mutants and the long-lived mitochondrial mutants *nuo-6* and *isp-1* are dependent on DAF-16 for their enhanced longevity (Senchuk et al., 2018; Dues et al., 2019). We also showed that loss of *tbc-2* decreases the lifespan of long-lived *daf-2* insulin-IGF1 receptor mutants (Meras et al., 2022). Since deletion of *tbc-2* affects the ability of DAF-16 to translocate to the nucleus (Traa et al., 2022), we wondered whether the disruption of *tbc-2* would also decrease the lifespan of the long-lived mitochondrial mutants. Accordingly, we crossed *tbc-2* deletion mutants to *nuo-6* and *isp-1* worms and quantified the resulting effect on lifespan. We found that *nuo-6* and *isp-1* worms both exhibit a marked decrease in lifespan when *tbc-2* is disrupted (Figure 1).

### Disruption of TBC-2 decreases resistance to specific stresses in long-lived mitochondrial mutants

Next, we sought to determine the role of TBC-2 in the enhanced stress resistance of the long-lived mitochondrial mutants and whether this might be contributing to the effect of *tbc-2* disruption on their lifespan. We quantified resistance to chronic oxidative stress (4 mM paraquat), acute oxidative stress (300 µM juglone), heat stress (37°C), bacterial pathogen stress (*P. aeruginosa* strain PA14), osmotic stress (450 mM and 550 mM NaCl) and anoxic stress.

**FIGURE 2:**
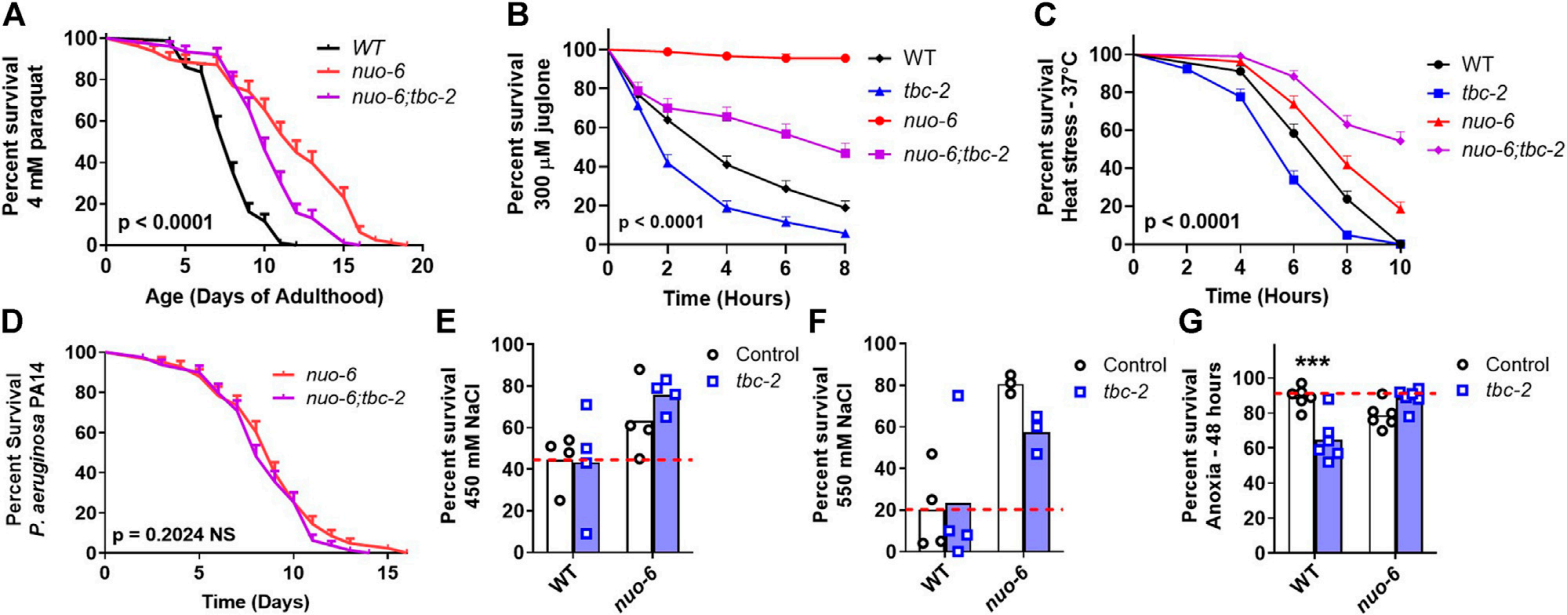
Disruption of *tbc-2* decreases resistance to oxidative stress in long-lived *nuo-6* mutants. Disruption of *tbc-2* decreases resistance to both chronic **(A)**; 4 mM paraquat) and acute **(B)**; 300 µM juglone) oxidative stress in *nuo-6* worms but has no significant effect on resistance heat stress **(C)**; 37°C) in these worms. Disruption of *tbc-2* did not affect resistance to bacterial pathogen stress **(D)**; *Pseudomonas aeruginosa* strain PA14), osmotic stress **(E, F)**; 450 mM NaCl, 550 mM NaCl) or anoxia **(G)**; 48 h) in *nuo-6* worms. A minimum of three biological replicates were performed. Statistical significance was assessed using the log-rank test in panels **(A–D)**. In panels **(A–D)**, *p*-values indicate the significance of difference between *nuo-6* (red line) and *nuo-6;tbc-2* (purple line). Significance was determined using a two-way ANOVA with Šidák’s multiple comparison test in panels **(E–G)**. Full statistical analyses can be found in Supplementary Table S1. Error bars indicate standard error (SE). ***p < 0.001.

**FIGURE 3:**
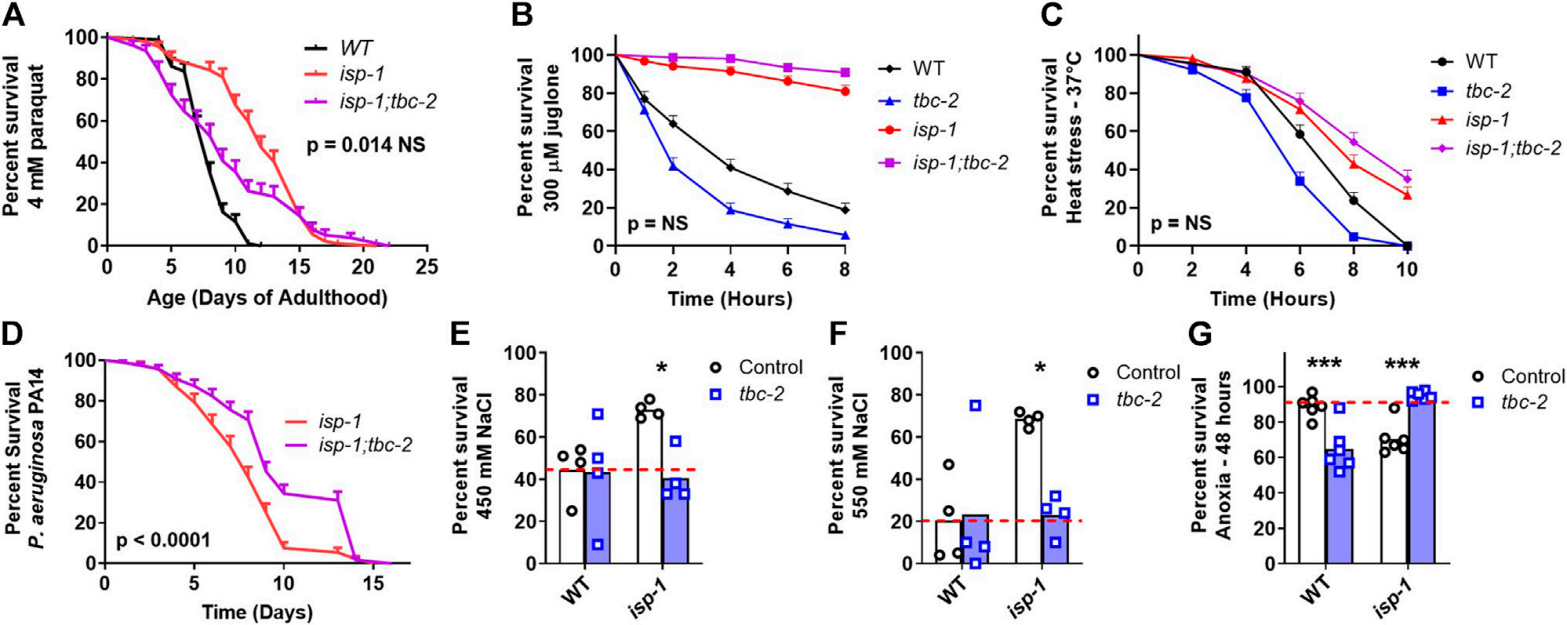
Disruption of *tbc-2* decreases stress resistance in long-lived *isp-1* mutants. Disruption of *tbc-2* decreases resistance to chronic oxidative stress in *isp-1* worms **(A)**; 4 mM paraquat) but has no effect on resistance to acute oxidative stress **(B)**; 300 µM juglone) or heat stress **(C)**; 37°C) in these worms. The *tbc-2* deletion increases resistance to bacterial pathogen stress in *isp-1* worms **(D)**; *P. aeruginosa* strain PA14). Disruption of *tbc-2* decreased resistance to osmotic stress in *isp-1* worms **(E, F)**; 450 mM NaCl, 550 mM NaCl), but resulted in an increased resistance to anoxia in *isp-1* mutants **(G)**; 48 h). Control data on wild-type and *tbc-2* mutants is repeated from Figure 2 for comparison. A minimum of three biological replicates were performed. Statistical significance was assessed using the log-rank test in panels **(A–D)**, and a two-way ANOVA with Šidák’s multiple comparison test in panels **(E–G)**. In panels **(A–D)**, *p*-values indicate significance of difference between *isp-1* (red line) and *isp-1;tbc-2* (purple line). Full statistical analyses can be found in Supplementary Table S1. Error bars indicate standard error (SE). *p < 0.05, ***p < 0.001.

In *nuo-6* mutants, disruption of *tbc-2* significantly decreased resistance to both chronic and acute oxidative stress (Figures 2A, B). In contrast, the loss of *tbc-2* did not significantly decrease resistance to heat stress (Figure 2C), bacterial pathogen stress (Figure 2D), osmotic stress (Figures 2E, F) or anoxic stress (Figure 2G) in *nuo-6* worms. In *isp-1* mutants, deletion of *tbc-2* decreased resistance to chronic oxidative stress (Figure 3A) but did not reduce resistance to acute oxidative stress (Figure 3B), heat stress (Figure 3C) or bacterial pathogens (Figure 3D). Disruption of *tbc-2* decreased resistance to osmotic stress in *isp-1* worms (Figures 3E, F), but did not decrease resistance to anoxia (Figure 3G). Combined, these results indicate that disruption of *tbc-2* alters resistance to some, but not all, external stressors in long-lived mitochondrial mutants, and primarily affects resistance to chronic oxidative stress.

### Disruption of TBC-2 does not decrease *isp-1* lifespan and resistance to oxidative stress in a DAF-16 null background

Our previous work suggests that the effect of TBC-2 disruption on stress resistance in wild-type and *daf-2* worms is primarily dependent on DAF-16, while its effect on wild-type and *daf-2* lifespan is also mediated by TBC-2’s impact on DAF-16-independent pathways (Traa et al., 2022). To gain insight into the extent to which deletion of *tbc-2* decreases oxidative stress resistance and lifespan through DAF-16-dependent pathways, we examined the effect of disrupting *tbc-2* in *isp-1* worms lacking DAF-16 (*isp-1;daf-16* mutants). We did not examine the DAF-16 dependency of the effect of *tbc-2* disruption on *nuo-6* worms because we are unable to generate *nuo-6;daf-16* or *nuo-6;daf-16;tbc-2* worms because of the close proximity of *nuo-6* and *daf-16* on the same chromosome.

**FIGURE 4:**
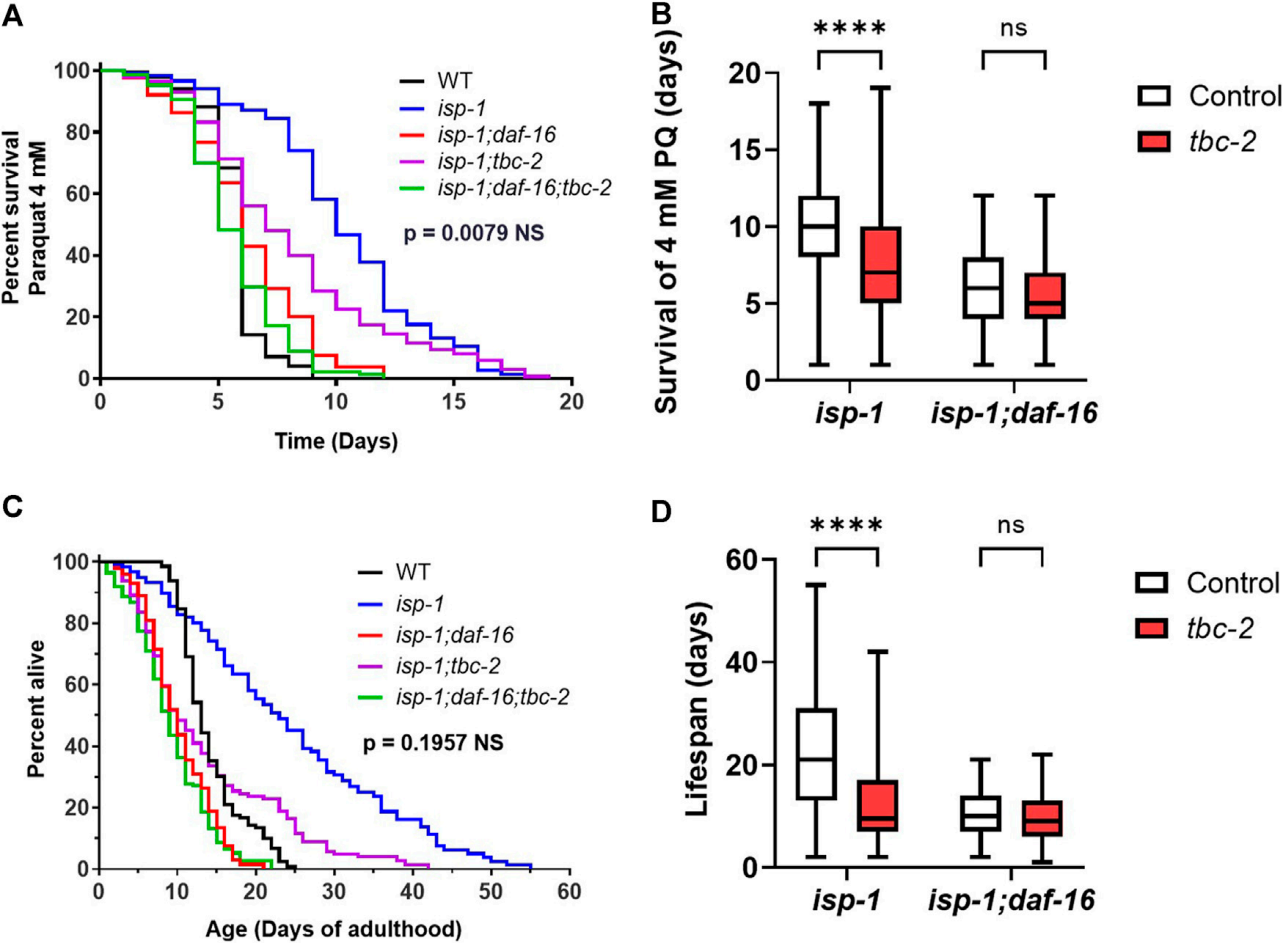
Loss of TBC-2 does not exacerbate the effects of disrupting DAF-16 on oxidative stress resistance or lifespan in *isp-1* mutants. To determine the role of DAF-16 in *tbc-2’s* detrimental impact on oxidative stress resistance and longevity, *tbc-2* was disrupted in *isp-1* worms with a deletion in *daf-16.* In *isp-1;daf-16* worms, deletion of *tbc-2* did not significantly decrease resistance to oxidative stress when exposed to 4 mM paraquat **(A, B)**. Similarly, disruption of *tbc-2* did not significantly decrease *isp-1;daf-16* lifespan **(C, D)**. In both cases, there was a trend towards decrease with the loss of *tbc-2.* Four biological replicates were performed. Statistical significance was assessed using a log-rank test in panels **(A, C)**, or a two-way ANOVA with Šidák’s multiple comparison test in panels **(B, D)**. In panels **(A, C)**, the *p*-value indicated is the significance of the difference between the *isp-1;daf-16*(blue line) and *isp-1;daf = 16;tbc-2* (purple line). Full statistical analysis can be found in Supplementary Table S1. Box plot error bars show minimum and maximum values. Box plot line indicates average value. NS = not significant, ****p < 0.0001.

We found that *isp-1;daf-16* mutants have markedly decreased resistance to oxidative stress (4 mM paraquat) compared to *isp-1* worms and that this deficit was not significantly exacerbated by disruption of *tbc-2* (Figures 4A, B). Similarly, *isp-1;daf-16* worms have a greatly reduced lifespan compared to *isp-1* worms and their lifespan is not further decreased by loss of *tbc-2* (Figures 4C, D). In both cases, however, there was a trend towards decrease in the absence of *tbc-2.*

### Disruption of TBC-2 does not prevent upregulation of DAF-16 target genes in long-lived mitochondrial mutants

In *daf-2* mutants, the nuclear localization of DAF-16 and the upregulation of DAF-16 target genes is inhibited by the loss of *tbc-2* (Meras et al., 2022). Similarly, TBC-2 is required for nuclear localization of DAF-16 in response to some stresses and for the full upregulation of DAF-16 target genes in wild-type worms exposed to exogenous stressors (Traa et al., 2022). Since the long-lived mitochondrial mutants also exhibit increased nuclear localization of DAF-16 and upregulation of DAF-16 target genes (Senchuk et al., 2018), we wondered whether TBC-2 is required for the increased expression of DAF-16 targets in these mutants and if the prevention of this gene upregulation might be contributing to the decrease in stress resistance and lifespan resulting from *tbc-2* deletion.

**FIGURE 5:**
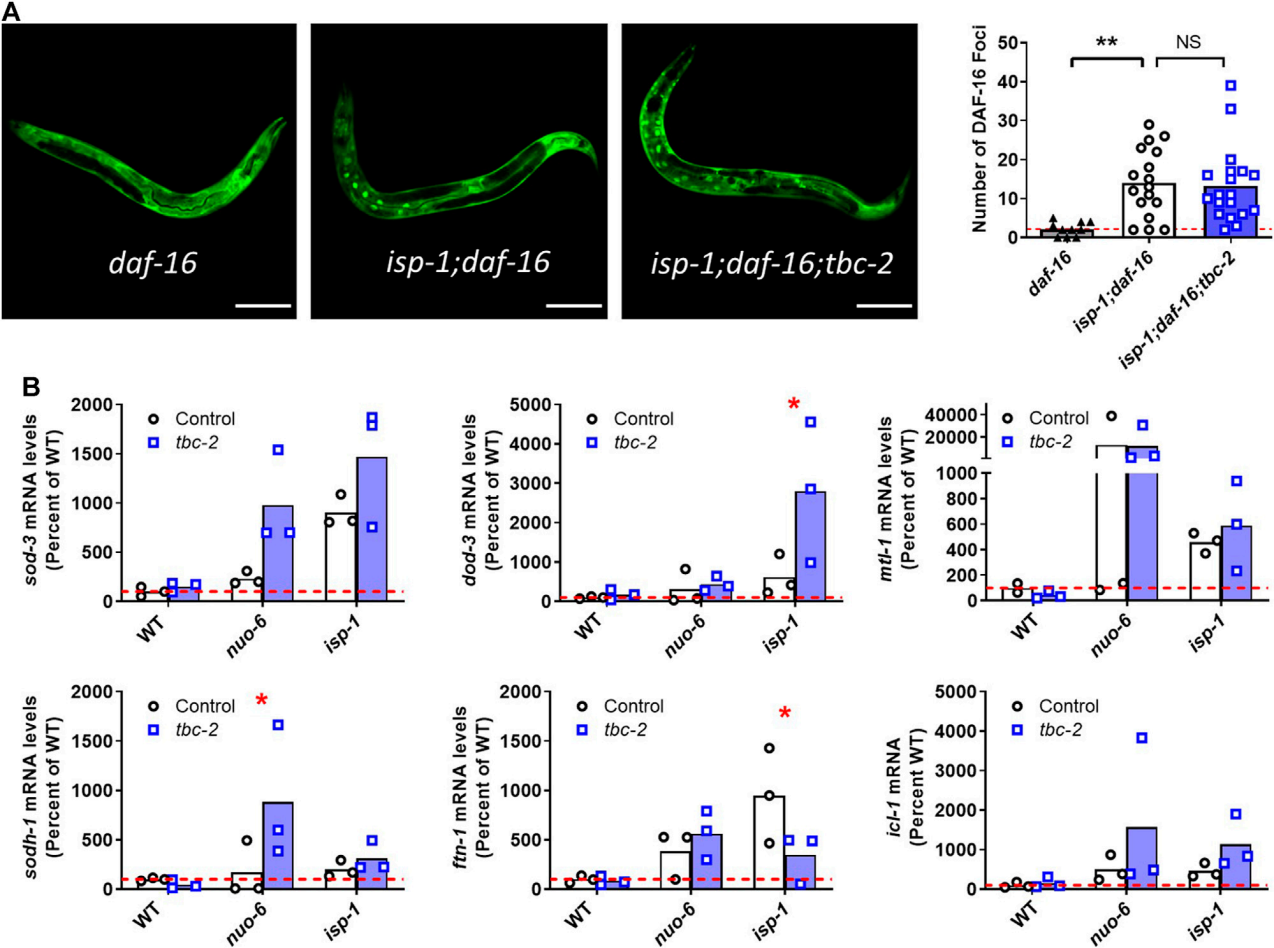
Disruption of TBC-2 does not affect nuclear localization of DAF-16 or prevent upregulation of DAF-16 target genes in long-lived mitochondrial mutants. **(A)** The nuclear localization of DAF-16 was assessed using zIs356[*daf-16p::daf-16::GFP*] worms. This experiment was performed in a *daf-16* mutant background because DAF-16 moves to the nucleus more readily than DAF-16::GFP. *isp-1* mutants have increased nuclear localization of DAF-16::GFP compared to wild-type worms. The nuclear localization of DAF-16 in *isp-1* worms was not affected by the disruption of *tbc-2.* Statistical significance was assessed using a one-way ANOVA with Dunnett’s multiple comparison test. **(B)** Quantitative RT-PCR was used to quantify the effect of *tbc-2* deletion on the upregulation of DAF-16 target genes (*sod-3, dod-3, mtl-1, sodh-1, ftn-1,* and *icl-1*) in the long-lived mitochondrial mutants, *nuo-6* and *isp-1*. All of the DAF-16 target genes were upregulated in *nuo-6* and *isp-1* mutants. In all but one instance, the disruption of *tbc-2* did not prevent the upregulation of DAF-16 target genes in the long-lived mitochondrial mutants. This suggests that TBC-2 is not required for the upregulation of DAF-16 target genes in the long-lived mitochondrial mutants. Three biological replicates were performed. Statistical significance was assessed using a two-way ANOVA with Šidák’s multiple comparison test. Full statistical analyses can be found in Supplementary Table S1. Error bars indicates SEM. *p < 0.05, **p < 0.01, ***p < 0.001.

To examine the effect of *tbc-2* disruption on the nuclear localization of DAF-16 in the long-lived mitochondrial mutants, we used a strain expressing DAF-16 linked to GFP under its endogenous promoter (zIs356[*daf-16p::daf-16::GFP*]). These experiments were performed in a *daf-16* mutant background, as previous studies have shown that it is important to remove the endogenous *daf-16* allele in order to properly visualize the nuclear localization of DAF-16::GFP likely because DAF-16 moves to the nucleus more readily than DAF-16::GFP (Putker et al., 2013; Senchuk et al., 2018). As we have observed previously, we found that *isp-1* worms have increased nuclear localization of DAF-16::GFP compared to wild-type worms (Senchuk et al., 2018). However, the increased nuclear localization of DAF-16 in *isp-1* worms was not affected by the disruption of TBC-2 (Figure 5A).

Next, we used quantitative RT-PCR to compare the expression of DAF-16 target genes between long-lived mitochondrial mutants in a wild-type and *tbc-2* mutant background. For this purpose, we chose six DAF-16 target genes (*sod-3, dod-3, mtl-1, sodh-1, ftn-1, icl-1*) that were among the top fifty DAF-16 upregulated genes identified by a meta-analysis of DAF-16 gene expression studies (Tepper et al., 2013), and which we have previously used to examine DAF-16 activity (Senchuk et al., 2018; Campos et al., 2021; Meras et al., 2022; Traa et al., 2022).

Consistent with our past results (Senchuk et al., 2018), we found that all six DAF-16 target genes are upregulated in both *nuo-6* and *isp-1* mutants (Figure 5B). Disruption of *tbc-2* did not result in a strong or consistent effect on the expression of DAF-16 target genes in the long-lived mitochondrial mutants. There was one example in which loss of *tbc-2* significantly decreased DAF-16 target gene expression (*ftn-1* levels in *isp-1* mutants) and two examples in which the loss of *tbc-2* resulted in a significant increase in the expression of DAF-16 target genes (*dod-3* in *isp-1* worms and *sodh-1* in *nuo-6* mutants). Disruption of *tbc-2* did not significantly alter the expression of most of the DAF-16 target genes examined in wild-type, *nuo-6* or *isp-1* worms (Figure 5B). Combined, this suggests that disruption of *tbc-2* does not markedly inhibit the ability of *nuo-6* and *isp-1* worms to upregulate DAF-16 target genes. However, it is possible that the upregulation of other DAF-16 target genes in *nuo-6* and *isp-1* worms is affected by *tbc-2* disruption.

## Discussion

What is the role of TBC-2 is stress resistance and lifespan? The results from our current and previous studies indicate that stress responsive factors such as the FOXO transcription factor DAF-16 can be localized to endosomes and that this localization is affected by TBC-2. Under normal conditions a small amount of DAF-16 is present on endosomes, while disruption of *tbc-2* results in increased levels of endosomal DAF-16 (Meras et al., 2022). When we originally observed DAF-16 on endosomes, we hypothesized that the increase in endosomal DAF-16 might allow for a stronger DAF-16 response to stress because of the larger stockpile of DAF-16 at the endosomes. However, it was also possible that disruption of TBC-2 would cause a weaker DAF-16 response because DAF-16 was stuck to endosomes. It turns out that both may be true depending on the stress. For heat stress, bacterial pathogen stress and anoxia, the nuclear localization of DAF-16 was slower in *tbc-2* mutants than wild-type worms, suggesting that endosomal DAF-16 is less able to move to the nucleus under these stresses (Traa et al., 2022). In contrast, DAF-16 moved to the nucleus more rapidly under chronic oxidative stress and osmotic stress in *tbc-2* mutants, suggesting that having a stockpile of DAF-16 at the endosomes enhances DAF-16 nuclear localization under these stresses.

In our current work, we find that the loss of TBC-2 decreases both stress resistance and lifespan in *nuo-6* mutants and *isp-1* mutants. We previously showed that disruption of *tbc-2* also decreases stress resistance and lifespan in long-lived *daf-2* mutants (Traa et al., 2022). Interestingly, the pattern of decreased stress resistance resulting from deletion of *tbc-2* differs between these three strains (Supplementary Table S2). While disruption of *tbc-2* decreases resistance to oxidative stress, heat stress, bacterial pathogen stress, osmotic stress and anoxia in *daf-2* mutants, resistance to heat stress, bacterial pathogen stress and anoxic stress is not decreased in either of the long-lived mitochondrial mutants when *tbc-2* is deleted. As our previous work suggests that the effect of *tbc-2* deletion on resistance to stress is primarily mediated by DAF-16 (Traa et al., 2022), it is plausible that stress resistance is more affected in *daf-2* mutants than *nuo-6* or *isp-1* mutants because the enhanced stress resistance of *daf-2* worms is entirely dependent on DAF-16 (Dues et al., 2019), while the long-lived mitochondrial mutants also rely on other pathways such as the mitochondrial unfolded protein response (Wu et al., 2018), the p38-mediated innate immune signaling pathway (Campos et al., 2021) and the mitochondrial thioredoxin system (Harris-Gauthier et al., 2022).

The only type of stress resistance that is significantly affected in all three strains is resistance to chronic oxidative stress. While this result is consistent with the possibility that disruption of *tbc-2* is decreasing lifespan by diminishing resistance to oxidative stress, the deletion of *tbc-2* also decreases lifespan in wild-type worms without affecting their ability to survive chronic oxidative stress (Traa et al., 2022). In addition, there are multiple examples in which resistance to oxidative stress can be experimentally dissociated from longevity (Van Raamsdonk and Hekimi, 2009; Van Raamsdonk et al., 2010; Shields et al., 2021).

To determine the extent to which deletion of *tbc-2* is decreasing oxidative stress resistance and lifespan through its effect on DAF-16, we examined the effect of disrupting *tbc-2* when DAF-16 is absent. We reasoned that if the *tbc-2* deletion is acting entirely through DAF-16 then disruption of *tbc-2* should not further decrease stress resistance or lifespan when DAF-16 is absent. We found that disruption of *tbc-2* resulted in a trend towards decreased resistance to chronic oxidative stress and lifespan in *isp-1;daf-16* mutants, which did not reach significance. This result is consistent with the effects of *tbc-2* deletion being mediated by DAF-16 but could also be explained by a floor effect since disruption of *daf-16* markedly reduces the lifespan and stress resistance of *isp-1* worms.

**FIGURE 6:**
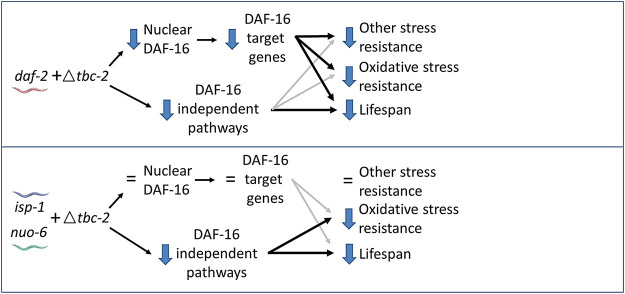
Model for contribution of DAF-16-dependent and DAF-16-independent pathways to effect of *tbc-2* deletion on stress resistance and longevity. In *daf-2* mutants, deletion of *tbc-2* decreases the nuclear localization of DAF-16 and decreases the expression of DAF-16 target genes. Decreased activation of DAF-16 is primarily responsible for the decreased stress resistance in *daf-2;tbc-2* mutants and partially contributes to their decrease in lifespan compared to *daf-2* worms. Decreased activation of DAF-16-independent pathways also contributes to the detrimental effect of *tbc-2* deletion on *daf-2* lifespan. In the long-lived mitochondrial mutants, *isp-1* and *nuo-6,* deletion of *tbc-2* does not affect the nuclear localization of DAF-16 or decrease the expression of DAF-16-target genes. Deletion of *tbc-2* also has little effect on resistance to most types of stress in *isp-1* and *nuo-6* worms. Disruption of *tbc-2* decreases chronic oxidative stress resistance and lifespan in *isp-1* and *nuo-6* worms primarily through DAF-16-independent pathways.

In exploring further, we found that disruption of *tbc-2* did not affect the nuclear localization of DAF-16 or prevent the upregulation of DAF-16 target genes in the long-lived mitochondrial mutants. These findings are consistent with the effects of *tbc-2* deletion being mediated by DAF-16-independent pathways (Figure 6). These differing conclusions may stem from the *daf-16* deletion having such a severe impact on *isp-1* stress resistance and longevity that the effects of *tbc-2* disruption on DAF-16-independent pathways are being masked. This possibility is supported by the fact that we did observe a trend towards decrease in *isp-1;daf-16;tbc-2* mutants compared to *isp-1;daf-16* mutants.

In contrast to the long-lived mitochondrial mutants, TBC-2 is required for the full upregulation of DAF-16 target genes in *daf-2* worms (Meras et al., 2022) and also important for the nuclear localization of DAF-16 in *daf-2* mutants (Traa et al., 2022). In these worms, the disruption of TBC-2 appears to be affecting stress resistance primarily through DAF-16 but affecting lifespan through DAF-16 and DAF-16-independent pathways (Traa et al., 2022). These differences likely stem from *daf-2* mutants being more dependent on DAF-16 for their stress resistance and longevity than the long-lived mitochondrial mutants, which rely more on DAF-16-independent pathways compared to *daf-2* worms. These DAF-16-independent pathways may include signaling through the nuclear hormone receptor NHR-49, epidermal growth factor signaling and notch signaling, all of which have been shown to be present on endosomes and influence longevity (Arantes-Oliveira et al., 2002; Wang et al., 2002; Curran and Ruvkun, 2007; Fortini and Bilder, 2009; Iwasa et al., 2010; Walter et al., 2011; Yu and Driscoll, 2011; Khan et al., 2013; Zheng et al., 2013; Dresen et al., 2015; Watterson et al., 2022). In the future, it will be important to assess the contribution of these and other signaling pathways that are present on endosomes in the long-lived mitochondrial mutants.

Two other endosome related proteins were recently shown to be required for the longevity of *isp-1* worms. After using proteomics to identify differentially expressed proteins in long-lived *clk-1* and *isp-1* mutants, a targeted RNAi screen was performed to assess the contribution of these proteins to their longevity. It was found that RNAi knockdown or genetic deletion of *mon-2* decreases *isp-1* lifespan (Jung et al., 2021). MON-2 regulates trafficking between endosomes and the Golgi apparatus (Mahajan et al., 2013). Similarly, TBC-3 is also involved in transport between endosomes and Golgi (Kanamori et al., 2008) and is required for *isp-1* lifespan (Jung et al., 2021). While MON-2 appears to be promoting longevity by upregulating autophagy, it is unclear how elevated endosomal MON-2 increases autophagy (Jung et al., 2021; Artan et al., 2022). Future studies will be needed to determine whether MON-2 and TBC-3 are required for endosomal signaling pathways, similar to TBC-2, or whether these endosome-related proteins are all acting through independent mechanisms.

## Conclusion

We find that disruption of the endosomal trafficking protein TBC-2 decreases oxidative stress resistance and lifespan in the long-lived mitochondrial mutants *nuo-6* and *isp-1.* Interestingly, the loss of TBC-2 does not prevent the upregulation of DAF-16 target genes in these mutants suggesting that the disruption of *tbc-2* may be affecting lifespan and resistance to stress in these worms by affecting other DAF-16-independent signaling pathways localized to endosomes. This work demonstrates an important role of endosomal signaling in stress resistance and lifespan.

## Data Availability

The original contributions presented in the study are included in the article/Supplementary Material, further inquiries can be directed to the corresponding author.
