# Supplementary material for: Endosomal trafficking protein TBC-2 is required for the longevity of long-lived mitochondrial mutants
Source: Front Aging. 2023 May 16;4:1145198. doi: 10.3389/fragi.2023.1145198 (PMC10228650; doi:10.3389/fragi.2023.1145198)
Supplement: Supplementary file 1 [file Table2.pdf]

Supplementary Table S2. Effect of TBC-2 disruption on stress resistance, gene expression and lifespan.

	Chronic Oxidative Stress	Acute Oxidative Stress	Heat Stress	Bacterial Pathogen Stress	Osmotic Stress	Anoxia	Expression of DAF-16 Target Genes	Lifespan
**WT**	No effect	Decreased	Decreased	Decreased	No effect	Decreased	No effect	Decreased
nuo-6	Decreased	Decreased	No effect	No effect	No effect	No effect	No effect	Decreased
isp-1	Decreased	No effect	No effect	Increased	Decreased	Increased	No effect	Decreased
daf-2	Decreased	No effect	Decreased	Decreased	Decreased	Decreased	Decreased	Decreased
